# The Impact of Chronic Oral Beta-Blocker Intake on Intravenous Bolus Landiolol Response in Hospitalized Intensive Care Patients with Sudden-Onset Supraventricular Tachycardia—A Post Hoc Analysis of a Cross-Sectional Trial

**DOI:** 10.3390/pharmaceutics16060839

**Published:** 2024-06-20

**Authors:** Felix Eibensteiner, Emmilie Mosor, Daniel Tihanyi, Sonja Anders, Andrea Kornfehl, Marco Neymayer, Julia Oppenauer, Christoph Veigl, Valentin Al Jalali, Hans Domanovits, Patrick Sulzgruber, Sebastian Schnaubelt

**Affiliations:** 1 Department of Emergency Medicine, Medical University of Vienna, 1090 Vienna, Austria; felix.eibensteiner@meduniwien.ac.at (F.E.);; 2 Department of Pulmonology, Clinic Ottakring, Vienna Healthcare Group, 1160 Vienna, Austria; 3 Department of Pulmonology, Clinic Penzing, Vienna Healthcare Group, 1140 Vienna, Austria; 4 Department of Clinical Pharmacology, Medical University of Vienna, 1090 Vienna, Austria; 5 Division of Cardiology, Department of Internal Medicine II, Medical University of Vienna, 1090 Vienna, Austria

**Keywords:** intensive care medicine, critical care, cardioselective β-blockers, landiolol, arrhythmia, dysrhythmia

## Abstract

**Background:** Landiolol, a highly cardioselective agent with a short half-life (2.4–4 min), is commonly used as a perfusor or bolus application to treat tachycardic arrhythmia. Some small studies suggest that prior oral β-blocker use results in a less effective response to intravenous β-blockers. **Methods:** This study investigated whether prior chronic oral β-blocker (Lβ) or no prior chronic oral β-blocker (L–) intake influences the response to intravenous push-dose Landiolol in intensive care patients with acute tachycardic arrhythmia. **Results:** The effects in 30 patients (67 [55–72] years) were analyzed, 10 (33.3%) with and 20 (66.7%) without prior oral β-blocker therapy. Arrhythmias were diagnosed as tachycardic atrial fibrillation in 14 patients and regular, non-fluid-dependent, supraventricular tachycardia in 16 cases. Successful heart rate control (Lβ 4 vs. L– 7, *p* = 1.00) and rhythm control (Lβ 3 vs. L– 6, *p* = 1.00) did not significantly differ between the two groups. Both groups showed a significant decrease in heart rate when comparing before and after the bolus administration, without significant differences between the two groups (Lβ −26/min vs. L– −33/min, *p* = 0.528). Oral β-blocker therapy also did not influence the change in mean arterial blood pressure after Landiolol bolus administration (Lβ −5 mmHg vs. L– −4 mmHg, *p* = 0.761). **Conclusions:** A prior chronic intake of β-blockers neither affected the effectiveness of push-dose Landiolol in heart rate or rhythm control nor impacted the difference in heart rate or mean arterial blood pressure before and after the Landiolol boli.

## 1. Introduction

Landiolol belongs to the group of ultra-short-acting, highly cardioselective and intravenously administered β-blockers [1]. It has a half-life of less than four minutes [2,3] and has a significantly stronger β-blocking effect and significantly higher cardioselectivity than the other well-known short-acting β-blocker Esmolol [4]. The advantage of Landiolol is considered to be its significantly higher cardioselectivity, which leads to significantly less negative inotropy and, thus, significantly less hypotension, which is an important safety aspect, especially in emergency medicine and critical care [5]. Some studies even describe an improvement in inotropy with Landiolol [6]. Landiolol is available both for continuous administration and as a bolus medication. Because critical care patients are often hemodynamically unstable, and as the drug has advantages over other β-blockers or rate- or rhythm-controlling drugs due to its good controllability (rapid onset of action, rapid loss of effect after cessation), continuous administration was primarily used in intensive care units in the past [7,8]. A potential problem in intensive care units is posed by not only tachycardic arrhythmia but also the sympathetic overstimulation caused by critical illness, which leads to an increased endogenous release of catecholamines [9,10]. This release (often additionally potentiated by exogenous catecholamine support) has a negative impact on outcomes and, in particular, cardiac function by stimulating β-receptors, leading to increased inotropy and chronotropy with increased myocardial oxygen consumption, and cardiomyocyte remodeling with an impact on left ventricular ejection fraction [11,12,13]. A recent meta-analysis by Heliste et al. showed a long-term survival benefit for critically ill patients with β-blocker treatment in a mixed intensive care population of more than 2000 patients [14].

However, Landiolol has recently also become increasingly appreciated as a bolus medication. This has the advantage of a faster onset of action compared with continuous administration [3]. However, if longer effectiveness over several days is required, continuous infusion still offers advantages over bolus administration.

Data on oral β-blockade prior to intravenous administration of a cardioselective β-blocker are scarce. In one study, bolus administration of Landiolol vs. other β-blockers/calcium antagonists in patients who had recently received oral β-blockers during computed tomography coronary angiography did not show a significantly different decrease in heart rate. However, the patients in both groups had only a very low proportion of long-term oral β-blocker medications [15]. In a small open-label study comparing Landiolol as either a continuous infusion or a bolus followed by an infusion in hemodynamically stable patients, patients with pre-existing oral β-blockade, in particular, failed to meet the primary endpoint of successfully lowering heart rate [16].

It has not been studied whether pre-existing oral β-blockade affects the effect of bolus Landiolol in critically ill intensive care patients.

## 2. Materials and Methods

### 2.1. Study Design

This study is a retrospective analysis of a cross-sectional study [17] that analyzed patients who received a bolus application of Landiolol for the treatment of sudden-onset tachycardic arrhythmia during their stay in an intensive care unit. The administration of Landiolol was based on the clinical decision of the attending physician. The patients were treated at different internal intensive care units in Vienna, Austria (Department of Pulmonology, Clinic Penzing, Vienna Healthcare Group and the Department of Emergency Medicine, Medical University of Vienna). All patients who developed tachycardic arrhythmia during their intensive care unit (ICU) stay and therefore received Landiolol as an antiarrhythmic or rate-controlling drug were included in a retrospective registry, which was used to conduct this post hoc analysis. This retrospective register was approved by the ethics committee (N°1442/2021).

### 2.2. Inclusion and Exclusion Criteria

*Inclusion:* We included all patients over 18 years of age who had received Landiolol due to sudden-onset, non-compensating supraventricular tachycardia. Moreover, we screened prior medication for oral β-blocker intake and other chronic medication intake.

*Exclusion:* We excluded patients below 18 years of age and those missing continuous invasive arterial pressure monitoring (IBPM) and heart rate measurements.

### 2.3. Data Collection

All patients who met the inclusion criteria and received Landiolol between August 2020 and October 2021 were included in the analysis. The data from the first bolus of Landiolol per patient were used for the analysis, and all subsequent boli (up to 4 boli) were excluded from further analysis, as a possible influence on β-receptors from the previous bolus of Landiolol could not be completely ruled out. These data had already been checked for the availability of invasive blood pressure measurement data in the previous study. Further data (e.g., demographics, previous illnesses, medication, vital parameters, effects of Landiolol) were collected from the intensive care unit’s data management system of each hospital. Oral β-blocker use was defined as existing prior medication in the current medication list and the re-establishment of β-blocker therapy during hospitalization prior to Landiolol administration. Due to the retrospective nature of the study, it was difficult to determine the exact duration of oral β-blockade. Some patients were unconscious at ICU admission. However, by reviewing the patients’ past medical histories, pre-existing drug prescriptions, and pharmacy records (in Austria, distributed medications in pharmacies are electronically registered with the last hand-over date) and talking to relatives, it was attempted to collect the respective information. Oral β-blocker medication was stopped at ICU admission and only started in the normal ward or after hospital discharge again.

### 2.4. Study Outcomes

The primary outcome parameter was the difference in counts of successful rate control after the bolus administration of Landiolol between patients with (Lβ) and without previous chronic oral β-blocker therapy (L–). The European Society of Cardiology (ESC) guidelines on atrial fibrillation state that the target heart rate is not entirely clear when aiming for rate control. However, a heart rate below 110/min should be aimed for [18]. The American Heart Association (AHA) also recommends a target heart rate of <100–110/min for patients without heart failure [19]. A low heart rate is recommended for patients with chronic heart failure or other cardiovascular comorbidities [20]. The difficulty of a uniform definition of heart rate control is also shown in a large meta-analysis by Johnston et al., where only 2 of 11 analyzed studies describe a heart rate <110/min as a successful HR reduction; the other data varied from study to study (e.g., >30% vs. >20%, <100/min vs. 69–94/min) [21]. In earlier studies investigating the effect of Landiolol on heart rate control, a 20% [22,23,24] or 10% [25] decrease in heart rate was rated as successful heart rate control. Due to these varying definitions, in our study, a heart rate reduction of >15% for at least 20 min was considered a successful heart rate reduction.

As a secondary outcome, we investigated the effect of Landiolol in terms of rhythm control (successful rhythm control was defined as a conversion to sinus rhythm for a period of at least 20 min), as well as the effects on heart rate and mean arterial blood pressure between patients on chronic oral β-blocker therapy (Lβ) and those without chronic oral β-blocker therapy (L–).

### 2.5. Statistical Analysis

Data were first tested using the Shapiro–Wilk test to verify the normal distribution of the parameters. Numerical data are presented as means ± standard deviations (SDs) or medians and the respective interquartile ranges (IQRs). Categorical variables are expressed as absolute and relative frequencies and as percentages of the total. The Fisher exact test was used to compare categorical parameters. The continuous data were compared using the unpaired *t*-test or the Kruskal–Wallis test. 

The Fisher exact test was used to compare L– and Lβ with respect to successful frequency control. To answer the secondary research questions, a Fisher exact test was used to compare rhythm control, and a paired *t*-test was used to compare mean arterial blood pressure and heart rate before and after bolus administration. In addition, an independent *t*-test was used to distinguish the mean change in heart rate and MAP between the two groups. We assumed statistical significance using two-sided *p*-values of <0.05. Calculations were performed using SPSS 27.0 (IBM, Armonk, New York, NY, USA).

## 3. Results

### 3.1. Data Distribution 

Except for age and the dosage of bolus administration, most data were normally distributed.

### 3.2. Patients

**Figure 1:**
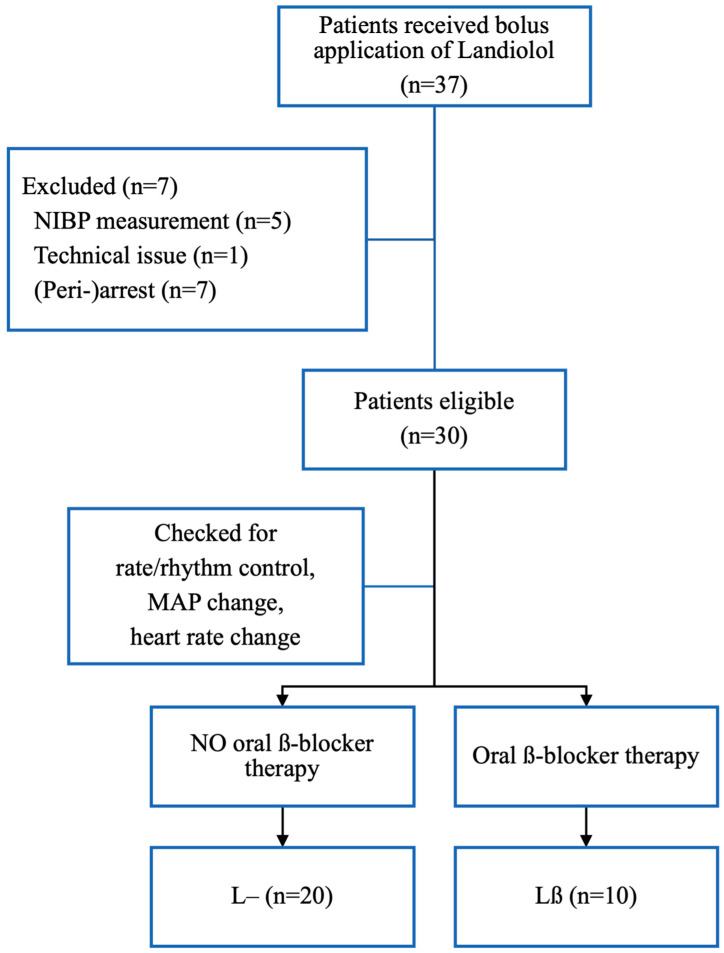
**Flowchart for study inclusion.** Lβ = existing oral β-blocker therapy before administration of Landiolol, L– = no existing oral β-blocker therapy before administration of Landiolol, NIBP = non-invasive blood pressure measurement.

A total of 30 patients with sudden-onset supraventricular tachycardia were included. Ten (33.3%) of these had been on long-term oral β-blocker therapy, and twenty (66.7%) had not (see Figure 1). Exactly nine (90%) in the Lβ group received Bisoprolol, and one patient received Nebivolol (10%). The mean age was 62.8 years (Lβ 68 ± 18 years vs. L 67 ± 13.2 years). Four (40%) women were included in the Lβ group and nine (45%) women in the L group.

A total of 8 out of 30 patients (26.7%) had no pre-existing medical conditions at the time of hospitalization. Further details regarding pre-existing medical conditions and the reason for ICU admission are shown in Table 1. The demographic parameters were homogeneously distributed between the groups, and there were no statistical differences.

### 3.3. Bolus Application of Landiolol

The average dose of push-dose Landiolol administered was 7.0 mg (6.5–11.0) (L– 7.0 mg [7.0–9.3] vs. Lβ 7.0 mg [6.0–11.0]; *p* = 0.779). In 14 patients (46.7%), the detected arrhythmia was tachycardic atrial fibrillation, and in 16 patients (46.7%), non-volume-dependent rhythmic supraventricular tachycardia (e.g., atrial flutter or atrial tachycardia). There were no differences between groups concerning catecholamines (dobutamine or noradrenaline), steroids, or antibiotics given at the time of bolus administration. Further details are described in Table 2.

### 3.4. Primary and Secondary Endpoints

All patients achieved one of the following three outcomes: rate control, rhythm control or neither. As a primary outcome, we compared the effect of Landiolol on heart rate between patients with pre-existing oral β-blockade (Lβ) and without pre-existing oral β-blockade (L–). In 11 patients (36.7%) out of 30, successful and sustained rate control was achieved shortly after bolus administration. No significant differences in successful rate control could be shown between the two groups Lβ vs. L– (4 vs. 7, *p* = 1.000). 

Successful rhythm control was also achieved in nine (30%) cases, with no significant difference between Lβ and L– (3 vs. 6, *p* = 1.000). Overall, Landiolol had a cumulative effect (rate and rhythm control) on acute supraventricular tachycardia in 20 patients (66.7%). See Table 3 for details.

#### 3.4.1. Effect of Bolus Landiolol on Blood Pressure

There was no significant decrease in blood pressure in either group (Lβ vs. L–) when comparing the mean arterial pressure (MAP) 15 min before bolus administration and 15 min thereafter. However, there was a tendency toward lower mean arterial pressure when comparing pre- vs. post-bolus administration (*p* = 0.080 in Lβ, *p* = 0.104 in L–). In the whole cohort, a significant but not clinically relevant decrease in mean arterial blood pressure was observed (*p* = 0.019). There was no significant effect of pre-existing β-blockade on the change in MAP after Landiolol administration (*p* = 0.761). Details are shown in Table 4 and Table 5.

#### 3.4.2. Effect of Bolus Landiolol on Heart Rate

A significant decrease in heart rate after the bolus application was observed in both groups Lβ (139 vs. 113 bpm, *p* = 0.003) and L– (146 vs. 112 bpm, *p* = < 0.001; Table 4), as well as in the two groups combined (143 vs. 112 bpm, *p* < 0.001; Table 4). However, there was no significant difference in the mean decrease in heart rate between the two groups (−26 vs. −33 bpm, *p* = 0.528) and therefore no indication in this patient population that prior β-blocker intake had had an impact on the effect of Landiolol (Table 5).

## 4. Discussion

**Figure 2:**
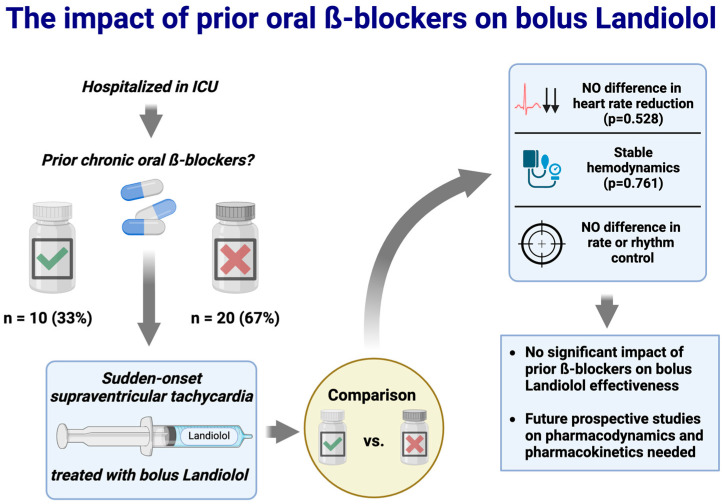
**Study overview.** ICU = intensive care unit. Created with BioRender.com.

In this study, we were able to show that prior administration of oral β-blockers had no significant impact on rate or rhythm control after Landiolol bolus administration (Figure 2 sums up the study findings). As already described in our initial cross-sectional study [17], Landiolol appears to have a high potential for successful rate and rhythm control in critically ill patients with a sudden onset of tachyarrhythmia (rate control in 11 and rhythm control in 9 patients). However, in one-third of patients, there was no effect. Landiolol has already shown a high potential for rhythm- and frequency-controlling effects in several studies in the ICU setting [7,8,21,22,26,27,28,29]. Compared with the frequently used amiodarone in intensive care medicine, Landiolol also showed a significantly faster rhythm-controlling effect in postoperative atrial fibrillation but with similar effectiveness in terms of rhythm control rates [30]. A very small study by Krumpl et al. was able to show that, in healthy subjects, Landiolol had a significantly faster onset of action and a longer-lasting effect than Esmolol, with a similar effect on blood pressure [31].

Thus, the idea behind our research question was that the majority of β1 receptors could already be occupied or partially occupied by previous oral β-blocker use (in most cases, this would be oral cardioselective β-blockers such as Bisoprolol, Metoprolol or Nebivolol), and therefore, the effect of a highly cardioselective β-blocker such as Landiolol could be attenuated. This is why we wanted to investigate the clinical effect of oral β-blockade on the efficacy of Landiolol in our small study, and why the theory of occupied β-receptors seems plausible. To the best of our knowledge, our study was the first study to investigate this question in a real-world clinical cohort. Only a few studies have so far reported on the combination of previously administered β-blockers, and mostly only in simulation models or healthy patient populations [15,16,32].

A small study by de Mey et al. in 1993 investigated the effect of Celiprolol, a cardioselective β1 antagonist and partial β2 agonist with a half-life of 4–5 h, in combination with Bisoprolol or placebo in healthy subjects [32]. This study showed that approximately 2.5 h after Bisoprolol administration, 78% of β1 receptors were occupied, and approximately three hours after Celiprolol administration, 88% of β1 receptors were occupied. However, it was shown that β1-receptor occupancy did not increase significantly between Bisoprolol + Celiprolol (90%) and placebo + Celiprolol (88%). Thus, the combination of two cardioselective β1-blockers did not increase β1-blockade, which may be explained by the fact that a large proportion of the receptors, 88%, were already occupied after Bisoprolol administration, and thus, the supposed maximum effect was almost reached. However, an increased significant reduction in heart rate was observed with Bisoprolol + Celiprolol vs. placebo + Celiprolol [32]. As we do not know the exact time between the last administration of oral β-blockers and Landiolol in our study, we could not measure β-receptor occupancy, and as we often use other drugs acting on β-receptors, like catecholamines, in the intensive care setting, we could not show this slight effect in the heart rate decrease in our patient population. 

However, there was another open-label pilot study in which Landiolol was investigated for the treatment of atrial fibrillation or flutter in cardiorespiratory stable patients: In Stix et al., 20 patients were included, and Landiolol was investigated with continuous administration or bolus application prior to continuous administration. The primary endpoint of the study was a sustained heart rate reduction (heart rate reduction >20% or <90/min within 16 min) in both groups. The effect of oral β-blockade on the achievement of the primary endpoint (heart rate control) was investigated as a secondary endpoint. It was shown that patients with oral β-blockade, pre-existing arterial hypertension, and pre-existing low systolic blood pressure in particular failed to reach the endpoint of heart rate control [16].

Another interesting aspect that may contribute to a better understanding of the β1-blocking effect is the receptor binding curves of different β-blockers (Propranolol, Esmolol, Landiolol, Metoprolol and Atenolol): In a study by Fujito et al., five different β-blockers and their effects on heart rate and forced expiratory volume in one second (FEV1) as a parameter for potential adverse effects were analyzed [33]. Theoretical analyses were used to calculate the respective reuptake binding curves, effects on heart rate and FEV1. Landiolol showed a strong reduction in heart rate (13.5%) and only a minor negative effect on FEV1. The receptor binding curves for Metoprolol or Atenolol, as an example of orally available β-blockers, showed that almost 80% of the receptors were still blocked after approximately 2–2.5 h, whereas with Landiolol, no β1 receptors were blocked after almost 30 min. The β2-blocking effect was also significantly longer-lasting with all other β-blockers than with Landiolol, which, in turn, suggests a significantly lower negative respiratory effect due to the short occupation of the β2 receptors in low concentrations [33]. Especially in a critically ill patient population, the aspect of prolonged β1-blockade in the acute setting can be a disadvantage, as in the event of adverse side effects, the prolonged effectiveness results in poorer controllability. Due to these aspects, Landiolol can be advantageous here due to its already-mentioned very short half-life. 

The degree of β1-receptor occupancy also appears to be dose-dependent. For example, the occupancy of β1 receptors increases after Bisoprolol administration from 24% at 0.625 mg to 71.6% at 5 mg [34]. This effect may be desirable in the context of chronic diseases (e.g., myocardial infarction, heart failure, hypertension) but is not always desirable in the acute setting.

Due to the retrospective nature of our study and the lack of a detailed record of the last administration of oral β-blockers, we were unable to determine the exact time between the last administration and the bolus administration of Landiolol. However, previous studies have shown that with a single dose of Bisoprolol, over 80% of β1 receptors were still occupied after 24 h, and over 50% were still occupied after 48 h [35]. 

As the length of hospital stay before bolus administration ranged from 1 to almost 50 days, the last intake of oral β-blockers could not be determined precisely. In patients who received the bolus only a few days after admission and in patients who had been in the hospital for a longer period, only the approximate time (12–24 h) of the last administration could be determined from the respective documentation. Moreover, none of our patients received the potent β1 agonist dobutamine, and therefore, β1-receptor occupancy can be excluded as a relevant factor.

Possible discussion points as to why Landiolol showed differences in effectiveness concerning the heart-rate-lowering effect in our population compared to the other studies could be the following.

The majority of our patients (n = 9) in the Lβ group had Bisoprolol as a prior therapy, and, as other studies have already shown, Bisoprolol already occupies > 80% of the receptors very quickly and for a long time [32,34]; therefore, we would assume here that Landiolol (in combination with Bisoprolol) would have a significantly improved effectiveness in lowering the heart rate compared to Landiolol without prior oral β-blocker therapy (similar to the study by de May et al. with Celiprolol in healthy subjects) [32]. 

Potentially, the complexity and variability of the clinical gestalt in the ICU (respiratory insufficiency, st.p. COVID, st.p. CPR, etc.) should not be ignored in the context of this study, as critically ill patients often experience increased sympathetic activation, which increases the release of endogenous catecholamines, and critical illness also often leads to exogenous catecholamine intake [14]. This overload of both endogenous and exogenous catecholamines leads to an increase in the number of alpha and β receptors on various tissue surfaces [12]. It is known that the effect of β-blockers is strongest at high catecholamine levels and high receptor density on the cell surface [36]. More receptors are available for the binding of agonists or antagonists, and Landiolol potentially occupies many of the free receptors due to its rapid effectiveness despite the existing β-blockade by the previously taken β-blockers. 

Moreover, β-blockers act as competitive antagonists, which means that they compete with agonists (e.g., endogenous or exogenous catecholamines) for receptor binding. In situations of high catecholamine doses, they are able to displace β-blockers from the receptor. As a result, β1 receptors would again be increasingly occupied by agonists or remain unoccupied, whereupon reaching higher concentrations of Landiolol could displace catecholamines (adrenaline and noradrenaline) from the receptors or occupy the free ones [36,37]. Although only eight (27%) of our patients required catecholamine support, the sympathetic activation caused by environmental influences (e.g., noise, lightning [38]), mechanical ventilation, or infection and the resulting increase in endogenous catecholamine release in critically ill patients should not be ignored [14,39,40]. The possibly increased catecholamine concentrations in these critically ill patients may have led to Landiolol having a similar effect in both groups, as the previously administered oral β-blockers had already been displaced from the receptors, and therefore, the heart rate, affected by Landiolol, showed no significant difference in either group.

Our study has several limitations. First of all, the biggest limitation is certainly the very small group size, which represents a very heterogeneous group, without being able to draw conclusions about large intensive care cohorts. Second, since the exact time and dosage of the last β-blocker intake and the duration of oral β-blockade were not exactly known due to the lack of information in the retrospective registry, this aspect should be taken into account in future studies. Therefore, it seems reasonable to measure serum levels and the density of occupied receptors in patients on long-term oral β-blocker treatment in order to characterize the mechanisms and better predict the effects of intravenous push-dose Landiolol. Third, our group was also very heterogeneous in terms of arrhythmias. In the future, a clear distinction should be made between the different types of arrhythmias, such as atrial fibrillation, atrial flutter, atrial tachycardia, etc. Fourth, we only used the first bolus doses in the patients and not all of them, as in the original study. Due to the small number of patients and the possible β-blocking effect of the previous administration of Landiolol, we did not want to include any further influencing factors in the analysis. In the future, the influence of multiple bolus doses should also be investigated if sufficient power is available. Further, we arbitrarily defined a heart rate reduction of >15% for at least 20 min as successful.

## 5. Conclusions

This was the first study to investigate the influence of previous chronic oral β-blockade on the effectiveness of the highly cardioselective β-blocker Landiolol in critically ill intensive care patients. In this very small retrospective study, bolus administration of Landiolol was essentially unaffected by prior oral β-blocker therapy, and there was no difference in rate and rhythm control or hemodynamics between the groups with and without prior beta-blockade. Due to the small group size in this study, no generalization is possible, and therefore, in future prospective studies, the effect of oral β-blockers on intravenous β-blockers should be investigated in more detail in terms of pharmacodynamics and pharmacokinetics.

## Figures and Tables

**Table 1:** **Study population characteristics.** Categorical data are presented as counts and percentages, and continuous data as means and +/− standard deviations (SDs) or medians and interquartile ranges (IQRs). Categorical data were analyzed using Fisher’s exact test. Continuous data were analyzed using an unpaired *t*-test or a Kruskal–Wallis test. Lβ = existing oral β-blocker therapy before administration of Landiolol, L– = no existing oral β-blocker therapy before administration of Landiolol, BMI = body mass index, CPR = cardiopulmonary resuscitation, COVID-19 = coronavirus disease, ICU = intensive care unit.

	Total	Lβ	L–	*p*-Value
	n = 30	n = 10	n = 20	
Female, sex, n (%)	13 (43.3)	4 (40.0)	9 (45.0)	1.000
Age, years (IQR)	67 (55–72)	68 (49–73)	67 (58–72)	0.787
Height, m (SD)	1.72 ± 0.10	1.71 ± 0.07	1.72 ± 0.11	0.507
Weight, kg (SD)	84.2 ± 20.8	76.5 ± 17.7	88.1 ± 21.6	0.262
BMI, kg/m^2^ (SD)	29 ± 7	26 ± 6	30 ± 7	0.414
**Medical history**				
Hypertension, n (%)	12 (40.0)	5 (50.0)	7 (35.0)	0.461
Hyperlipidemia, n (%)	3 (10.0)	1 (10.0)	2 (10.0)	1.000
Diabetes, n (%)	10 (33.3)	4 (40.0)	6 (30.0)	0.690
Peripheral arterial disease, n (%)	2 (6.7)	0 (0.0)	2 (10.0)	0.540
Coronary artery disease, n (%)	6 (20.0)	1 (10.0)	5 (25.0)	0.633
Chronic kidney disease, n (%)	4 (13.3)	0 (0.0)	4 (20.0)	0.272
Atrial fibrillation, n (%)	13 (43.4)	4 (40.0)	9 (45.0)	1.000
Atrial flutter, n (%)	2 (6.7)	0 (0.0)	2 (10.0)	0.540
Thyroid disease, n (%)	8 (26.7)	3 (30.0)	5 (25.0)	1.000
**Reason for ICU admission**				
Neurological reason, n (%)	1 (3.3)	0 (0.0)	1 (5.0)	1.000
Dysrhythmia, n (%)	1 (3.3)	0 (0.0)	1 (5.0)	1.000
Respiratory failure, n (%)	10 (33.3)	4 (40.0)	6 (30.0)	0.690
Post-CPR, n (%)	3 (10)	0 (0.0)	3 (15.0)	0.532
Weaning post-COVID-19, n (%)	11 (36.7)	4 (40.0)	7 (35.0)	1.000
Sepsis, n (%)	2 (6.7)	1 (10.0)	1 (5.0)	1.000
Other reasons, n (%)	2 (6.7)	1 (10.0)	1 (5.0)	1.000

**Table 2:** **Characteristics at the time of bolus administration.** Categorical data are presented as counts and percentages, and continuous data as means and +/− standard deviations (SDs) or medians and interquartile ranges (IQRs). Categorical data were analyzed using Fisher’s exact test. Continuous data were analyzed using an unpaired *t*-test or a Kruskal–Wallis test. Lβ = existing oral β-blocker therapy before administration of Landiolol, L– = no existing oral β-blocker therapy before administration of Landiolol.

	Total	Lβ	L–	*p*-Value
	n = 30	n = 10	n = 20	
Dose of Landiolol, mg (IQR)	7 (6.5–11.0)	7.0 (6.0–11.0)	7.0 (7.0–9.3)	0.766
Hospitalization until bolus application, days (SD)	15 ± 18	15 ± 15	16 ± 19	0.894
Fluid balance at timeof bolus, mL (SD)	871 ± 1753	840 ± 1315	870 ± 1950	0.967
Potassium, mmol/L (SD)	4.1 ± 0.7	3.9 ± 0.4	4.1 ± 0.5	0.268
Magnesium, mmol/L (SD)	0.84 ± 0.21	0.79 ± 0.29	0.87 ± 0.17	0.428
Non-invasive or invasive ventilation at bolus application, n (%)	28 (93.3)	8 (80.0)	20 (100.0)	0.103
**Medication at time of bolus administration**				
Noradrenaline support, n (%)	8 (26.7)	1 (10.0)	7 (35.0)	0.210
Steroids, n (%)	12 (40.0)	4 (40.0)	8 (40.0)	1.000
Antibiotics, n (%)	25 (83.3)	7 (70.0)	18 (90)	0.300
Dobutamin, n (%)	0 (0)	0 (0)	0 (0)	-
**Tachycardia at time of bolus administration**				
Rhythmic, n (%)	16 (53.3)	5 (50.0)	11 (55.0)	1.000
Arrhythmic, n (%)	14 (46.7)	5 (50.0)	9 (45.0)	1.000

**Table 3:** **The arrhythmia-modulating effect of bolus Landiolol.** The effect of Landiolol on rate and rhythm within the cohort and Lβ and L–. Categorical data are presented as counts and percentages. Categorical data were analyzed using Fisher’s exact test. Lβ = existing oral β-blocker therapy before administration of Landiolol, L– = no existing oral β-blocker therapy before administration of Landiolol, MAP = mean arterial pressure, SD = standard deviation.

	Total	Lβ	L–	*p*-Value
	n = 30	n = 10	n = 20	
Rate control, n (%)	11 (36.7)	4 (40.0)	7 (35.0)	1.000
Rhythm control, n (%)	9 (30.0)	3 (30.0)	6 (30.0)	1.000
No effect, n (%)	10 (33.3)	3 (30.0)	7 (35.0)	1.000

**Table 4:** **The effect of Landiolol on mean arterial pressure and heart rate 15 min before and after the bolus within the whole study cohort and within the two groups Lβ and L–.** Data presented as means and +/− standard deviations. Data were analyzed using a paired *t*-test. Lβ = existing oral β-blocker therapy before administration of Landiolol, L– = no existing oral β-blocker therapy before administration of Landiolol, MAP = mean arterial pressure, SD = standard deviation, bpm = beats per minute.

Mean Arterial Pressure				
	Patients	Pre-Bolus	Post-Bolus	*p*-Value
MAP, mmHg (SD)	Total	97 ± 17	93 ± 19	**0.019**
	Lβ	101 ± 12	97 ± 15	0.080
	L–	95 ± 19	91 ± 20	0.104
**Heart rate**				
Heart rate, bpm (SD)	Total	143 ± 17	112 ± 24	**<0.001**
	Lβ	139 ±19	113 ± 20	**0.003**
	L–	146 ± 16	112 ± 27	**<0.001**

**Table 5:** **The effect of Landiolol on mean MAP and heart rate difference 15 min before and after the bolus between Lβ and L–.** Data presented as means and +/− standard deviations. Data were analyzed using an unpaired *t*-test. Lβ = existing oral β-blocker therapy before administration of Landiolol, L– = no existing oral β-blocker therapy before administration of Landiolol, MAP = mean arterial pressure, SD = standard deviation, bpm = beats per minute.

**MAP Difference 15 min Pre- and Post-Bolus**			
**Groups**	**Lβ**	**L–**	***p*-Value**
MAP difference, mmHg (SD)	−5 ± 8	−4 ± 10	0.761
**Heart Rate Difference**			
**Groups**	**Lβ**	**L–**	***p*-Value**
Mean difference in heart rate, bpm (SD)	−26 ± 21	−33 ± 34	0.528

## Data Availability

All data will be made available by the corresponding author upon reasonable request due to local law and regulations.
